# Long-Term Outcomes Over 20 Years in Persons With Persistent Disorders of Consciousness After Traumatic Brain Injury

**DOI:** 10.1089/neur.2023.0080

**Published:** 2023-11-21

**Authors:** Youhei Nakamura, Tadahiko Shiozaki, Hiroshi Ito, Shunichiro Nakao, Hiroshi Ogura, Jun Oda

**Affiliations:** Department of Traumatology and Acute Critical Medicine, Osaka University Graduate School of Medicine, Suita, Japan.

**Keywords:** Disability Rating Scale, long-term outcomes, traumatic brain injury, vegetative state

## Abstract

The long-term outcomes of patients with disorders of consciousness after traumatic brain injury (TBI) is unclear. We investigated the long-term outcomes over 20 years in patients who were in a persistent vegetative state (VS). We conducted a retrospective cohort study using a review of medical records and collected data by telephone and written interviews with patients and their families. We included patients who were treated for TBI at our hospital, between October 1996 and January 2003 and who were in a persistent VS, defined as a Disability Rating Scale (DRS) score of ≥22 at 1 month after TBI. The DRS was administered at 1 month, 6 months, 1 year, and then annually out to 20 years. We evaluated their clinical course until July 2021 with the DRS. We analyzed 35 patients in a persistent VS attributable to TBI. We were able to confirm the 20-year outcomes for 26 of the 35 patients (74%); at 20 years post-TBI, 19 (54%) patients were found to be deceased and 7 (20%) were alive. Over the 20-year study period, 23 of the 35 patients (65.7%) emerged from a persistent VS. Among the 35 patients in a persistent VS at 1 month post-TBI, 20 (57%) emerged from a persistent VS within 1 year, and 3 patients (8.6%) emerged from a persistent VS after more than a year after injury. DRS scores improved up to 9 years post-injury, whereas the change in DRS scores from 10 to 20 years post-injury was within ±1 point in all patients. We found that patients with persistent VS attributable to TBI may show improvement in functional disability up to 10 years post-injury. On the other hand, no substantial improvement in functional disability was observed after the 10th year.

## Introduction

The outcomes of persons with disorders of consciousness (DoC) attributable to traumatic brain injury (TBI) are often evaluated after 6 months to 1 year, and the long-term outcomes beyond 10 years is still unknown. A vegetative state (VS) that lasts for more than 1 month post-TBI is called a persistent VS, and that which lasts for more than 1 year post-TBI is called a chronic VS, which used to be called permanent VS.^1,2^ Typically, patients are expected to recover consciousness within 1 year of their injury, and it is considered less common to improve consciousness after 1 year.^3–5^ However, it has been reported that the outcomes of patients with TBI at 2 years is better than previously appreciated^6^ and also reported that late recovery after a traumatic long-lasting VS is not exceptional.^7,8^

Recent reports suggest that it may be in the patient's best interest to continue functional assessment and updated care plans for patients with DoC for at least 10 years post-TBI.^8^ However, long-term outcomes of patients with DoC ≥10 years after TBI has not been reported. We conducted this study to examine the hypothesis that functional recovery in patients with a persistent VS may continue beyond 10 years post-injury. If functional recovery continues beyond 10 years post-injury, this may suggest that continued functional assessment and care plan updates over the very long term may be beneficial to the patient. The objective of this study was to evaluate the long-term trajectory of outcomes using the Disability Rating Scale (DRS) of patients with TBI in a persistent VS over a 20-year period by retrospective data analysis.

## Methods

### Study design

We conducted a retrospective cohort study using a review of medical records and collected data by telephone and written interviews with patients and their families. We used the Strengthening the Reporting of Observational Studies in Epidemiology (STROBE) reporting guidelines in preparing this article.^9^ This study was approved by the ethics committee of Osaka University Hospital, which agreed with the opt-out consent process used (approval no. 21011).

### Setting

This study was conducted at the Department of Traumatology and Acute Critical Medicine, Osaka University Graduate School of Medicine, which is a tertiary care center. The center specializes in severe trauma and acute critical care and is staffed by neurosurgeons, trauma surgeons, emergency physicians, and intensive care physicians. It is equipped with computerized tomography (CT) scanning equipment and an operating room that enables the rapid diagnosis and treatment of TBI. The center has 20 intensive care beds for the provision of acute care, and once a patient's condition stabilizes, the patient is discharged home or transferred to another hospital for rehabilitation or recuperative treatment.

### Participants

All patients with TBI who were treated at the Department of Traumatology and Acute Critical Medicine, Osaka University Graduate School of Medicine, between October 1996 and January 2003, and who were in a VS based on the DRS at 1 month after admission, were included in this study. Dr. Nakamura (the corresponding author) and Dr. Shiozaki (the second author) reviewed the medical records. For patients discharged from the hospital within 1 month post-injury, Dr. Shiozaki visited the patient 1 month after their injury to evaluate and record the DRS. The diagnosis of TBI is based on the presence of intracranial hemorrhage, cerebral contusion, or diffuse axonal injury findings on head CT or a magnetic resonance imaging (MRI) scan. In this study, we diagnosed VS with a DRS score of ≥22 and defined a DRS of <22 as recovery from a VS. Patients who died within 1 month of admission, those who recovered from a VS within 1 month, and those younger than 15 years of age at admission were excluded.

### Variables

**FIG. 1.**
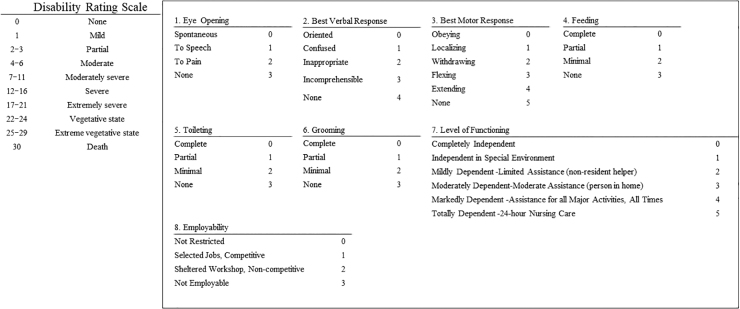
Specific contents of the Disability Rating Scale (DRS). The DRS consists of eight items (Eye Opening, Best Verbal Response, Best Motor Response, Cognitive Ability for Feeding, Toileting, Grooming, Level of Functioning, and Employability) and is used to assess the level of disability for severe traumatic brain injury. The DRS of 22–29 is defined as a vegetative state.

We evaluated patient background, Glasgow Coma Scale (GCS) on admission, injury mechanism, imaging findings including the Traumatic Coma Data Bank (TCDB) CT scan classification, length of stay in the hospital, and treatment details based on the medical records. We used the DRS as the indicator of long-term outcomes. The DRS consists of eight items (Eye Opening, Best Verbal Response, Best Motor Response, Cognitive Ability for Feeding, Toileting, Grooming, Level of Functioning, and Employability) and is used to assess the level of disability for severe TBI (Fig. 1). The DRS is used as an index to evaluate functional transition in patients with severe TBI^10^ and is reported to reflect the improvement effect of rehabilitation treatment more accurately and assess the clinical course of patients with severe TBI on a more sensitive scale than the Glasgow Outcome Scale,^11,12^ so we considered it useful as an index for evaluating long-term outcomes in this study.

In our study, the DRS was administered by trained and qualified emergency physicians or neurosurgeons at 1 month, 6 months, 1 year, and then annually until 2016, with the last administration in 2021. They evaluated the DRS in person or by telephone interviews with family members, caregivers, or medical social workers. For those without 20-year outcomes because of a loss to follow-up, we treated them as censored, whereas patients who died during the follow-up period and those whose DRS could be evaluated at the 20th year were treated as having completed follow-up. For each case, we evaluated whether the DRS follow-up outcomes were confirmed for all epochs, the duration of follow-up, the DRS trend, history of inpatients' rehabilitation treatment, and the cause of death. Patients with a DRS of ≥22 until the first year post-injury were defined as chronic VS and those with a DRS <22 were defined as non-chronic VS.

### Statistical analysis

Patient characteristics are reported using descriptive statistics. Continuous variables are presented as medians and interquartile range (IQR), and categorical variables are presented as counts and percentages. A line chart was used to visualize the trend in DRS over time. Data were analyzed using JMP Pro 16.2 statistical analysis software (SAS Institute Inc., Cary, NC).

## Results

**FIG. 2.**
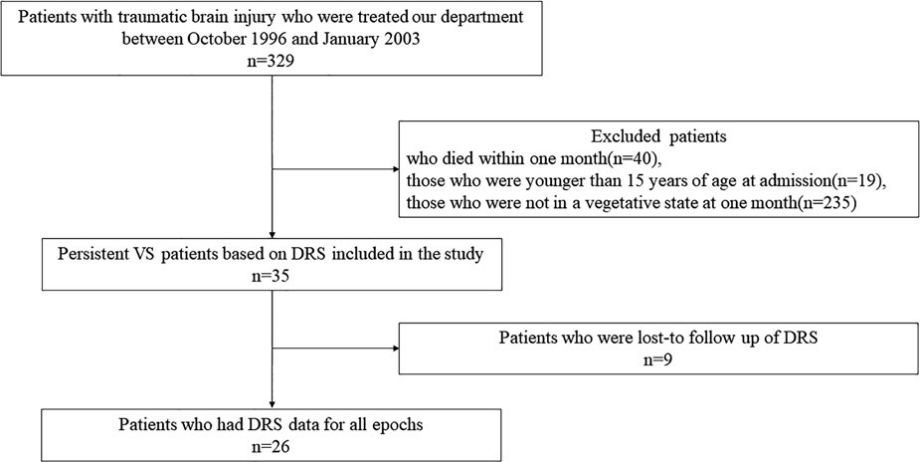
Patients chart flow. Of the 329 patients with traumatic brain injury, 35 were persistent VS cases with a DRS score of ≥22 at 1 month post-injury. Of these, 26 patients had DRS data available for all epochs. DRS, Disability Rating Scale; VS, vegetative state.

There were 329 patients with TBI subjected to chart review during the study period. Of these, 35 patients with a persistent VS at 1 month were identified through the review of medical records and were included in the study (Fig. 2). Their characteristics are shown in Table 1.

**Table 1.** Patient Characteristics

	All patients (*n* = 35)	Patients who had DRS data for all epochs (*n* = 26)	Chronic VS patients (*n* = 14)	Non-chronic VS patients (*n* = 19)
Age at admission, median (IQR), years	44 (26–60)	54 (26–66)	56 (29–63)	42 (26–55)
Sex, *n* (%)				
Male	27 (77.1)	20 (77)	11 (79)	14 (74)
Female	8 (22.9)	6 (23)	3 (21)	5 (26)
GCS at admission, median (IQR)	5 (4–7)	5 (4–7)	5 (3–6)	5 (4–10)
Injury mechanism, *n* (%)				
Traffic accident	24 (68.6)	16 (62)	8 (57)	16 (84)
Fall	8 (22.9)	8 (31)	4 (29)	2 (11)
Assault	2 (5.7)	0 (0)	1 (7)	1 (5)
Unknown	1 (2.9)	0 (0)	1 (7)	0 (0)
Imaging diagnosis, *n* (%)				
Contusion	27 (77.1)	22 (85)	14 (100)	17 (89)
Subarachnoid hemorrhage	19 (54.3)	13 (50)	9 (64)	8 (42)
Subdural hematoma	18 (51.4)	15 (58)	7 (50)	10 (53)
Epidural hematoma	6 (17.1)	4 (15)	1 (7)	5 (26)
Diffuse axonal injury	7 (20.0)	5 (19)	3 (21)	3 (16)
TCDB CT classification, *n* (%)				
Diffuse injury I	2 (5.7)	2 (8)	0 (0)	2 (11)
Diffuse injury II	4 (11.4)	2 (8)	0 (0)	3 (16)
Diffuse injury III	9 (25.7)	6 (23)	6 (43)	3 (16)
Diffuse injury IV	0 (0)	0 (0)	0 (0)	0 (0)
Evacuated mass	20 (57.1)	16 (62)	8 (57)	11 (58)
Non-evacuated mass	0 (0)	0 (0)	0 (0)	0 (0)
Surgical procedures, *n* (%)				
Craniotomy	15 (42.9)	11 (42)	4 (29)	10 (53)
Trepanation	9 (25.7)	4 (15)	5 (36)	4 (21)
Decompressive craniectomy	9 (25.7)	7 (27)	1 (7)	7 (37)
Tracheotomy	34 (97.1)	25 (96)	14 (100)	18 (95)
Hypothermia treatment, *n* (%)	20 (57.1)	16 (62)	9 (64)	11 (58)
Barbiturate therapy, *n* (%)	28 (80.0)	21 (81)	14 (100)	13 (68)
ICP monitoring, *n* (%)	32 (91.4)	23 (88)	13 (93)	17 (89)
Length of stay in our hospital, median (IQR), days	43 (27–61)	45 (27–65)	50 (40–69)	38 (27–61)
DRS at 1 month post-injury, median (IQR)	26 (24–27)	26 (24–27)	26 (25–27)	25 (23–26)

GCS, Glasgow Coma Scale; TCDB, Traumatic Coma Databank; CT, computed tomography; ICP, intracranial pressure; DRS, Disability Rating Scale; IQR, interquartile range.

All patients were transferred to other hospitals, and none were discharged home. After transfer from our hospital, 15 patients (43%) were found to be undergoing rehabilitation treatment. Of these, 2 (13%) were chronic VS patients and 13 (87%) were non-chronic VS patients.

The median DRS at 1 month after admission for the patients in this study was 26 (24–27). Among the 35 patients in a persistent VS at 1 month, we had complete DRS follow-up data at all years in 26 patients (74%), of whom 7 (20%) were alive and 19 (54%) had died. Nine patients (26%) were lost-to follow up and were treated censored patients. The median follow-up period for all 35 patients was 7 (4–17) years. The median follow-up period for the 26 patients who had DRS data for all epochs was also 7 (4–20) years, whereas that for the 9 censored patients was 13 (6–17) years (Table 2).

**Table 2.** Long-Term Prognostic Follow-up Results

	Total* n* = 35* n *(%)	Follow-up period median (IQR), years
Study patients	35 (100)	7 (4–17)
Patients with confirmed outcome up to 20 years	26 (74.3)	7 (4–20)
Survived	7 (20.0)	20
Died	19 (54.3)	4 (2–7)
Patients with follow-up censored	9 (25.7)	13 (3–18)
Patients in a chronic vegetative state	14 (40.0)	5 (3–8)

IQR, interquartile range.

The 26 patients who had DRS data for all epochs had a median survival of 7 (4–20) years.

We confirmed that 19 patients (54%) died in the study period, with 16 dying by the 10th year. Causes of death were pneumonia in 11, asphyxia in 1, pyelonephritis in 1, renal failure in 1, sepsis in 1, enteritis in 1, stroke in 1, and unknown in 2 patients.

**FIG. 3.**
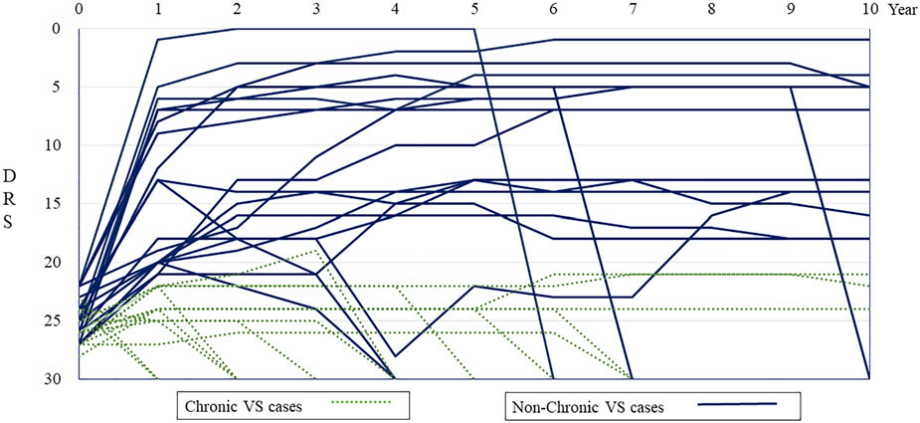
Changes in the DRS in the first decade of the 20-year period. Of the 35 patients with persistent VS, 20 (solid blue lines) recovered from a VS (DRS <22) in the first year, and the trend in DRS in some patients continued to improve. Two patients showed improvement in function to mild disability that allowed them to return to work. The remaining 15 cases (green dashed lines) were patients in a chronic VS. Of these 15 patients, 12 died within 10 years and only 3 recovered from a VS (DRS <22). DRS, Disability Rating Scale; VS, vegetative state.

**FIG. 4.**
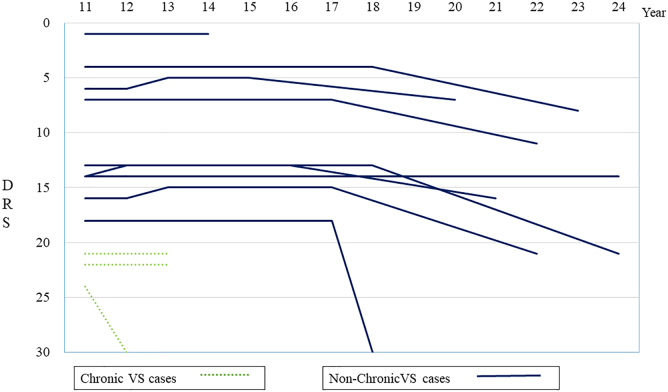
Changes in the DRS in the second decade of the 20-year period. After the first 10 years, no case improved more than 2 points in the DRS and little change occurred. DRS, Disability Rating Scale; VS, vegetative state.

The course of DRS during the first 10 years is shown in Figure 3. Twenty patients (54.1%) recovered from their VS by the first year after injury (non-chronic VS), but 14 patients (40%) did not and were considered to be in a chronic VS. One patient died within a year without recovering from VS. Of the non-chronic VS patients, 2 (10%) showed improvements in function to the point of mild disability that allowed them to return to work. In addition, up to the ninth year post-injury, there were patients in whom the DRS improved by ≥2 points. However, after 10 years of injury, all patients' DRS scores changed by within ±1 point (Fig. 4). Among the 20 patients who improved from a VS within 1 year post-injury, the DRS improved by ≥2 points after the first year in 13 patients (65%). However, among the 14 patients in a chronic VS, the DRS improved by ≥2 points after the first year of injury in only 2 (14%) patients. Although 3 chronic VS patients (21%) emerged from their VS during the study period, none who were in a chronic VS improved beyond severe disability. Twelve (86%) of the 14 chronic VS patients died (11 within the first 10 years), and none were alive at the 20th year.

**FIG. 5.**
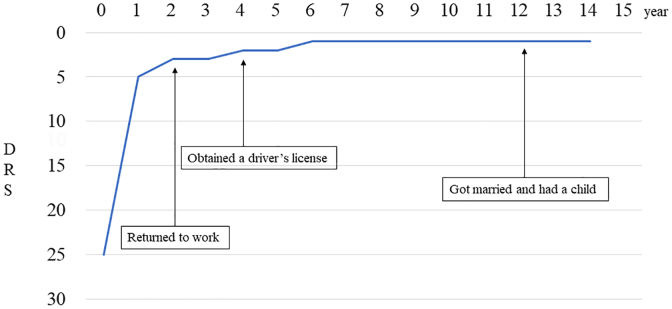
Changes in the DRS for case 1. Case 1 is a teenager with multiple trauma injuries from a traffic accident. The DRS at 1 month post-injury was 25; at 6 months post-injury, the DRS improved to DRS 5, and at 1 year post-injury, the DRS was still 5. The patient continued to show gradual functional recovery and returned to work with a DRS of 3 at 2 years post-injury. The patient continued to show functional recovery, improving to DRS 1 at 6 years post-injury and remaining at DRS 1 at 14 years. During this period, the patient was able to return to normal social activities, including driving a car and getting married. DRS, Disability Rating Scale.

We describe the progress of the 2 patients who returned to society. Case 1 was a teenage male suffering multiple trauma attributable to a traffic accident (Fig. 5). The patient's level of consciousness on hospital arrival was GCS E2V1M4, and head CT findings showed a left temporal lobe cerebral contusion and traumatic subarachnoid hemorrhage. Surgical treatment included ventricular drainage and tracheostomy. One month post-injury, his DRS was 25 and he was transferred to a rehabilitation hospital. Six months post-injury, he had improved to DRS 5. One year later, he was still at DRS 5, but continued to show gradual functional recovery and returned to work 2 years after the injury at DRS 3. The DRS scores were composed of Level of Functioning 1 and Employability 2.

**FIG. 6.**
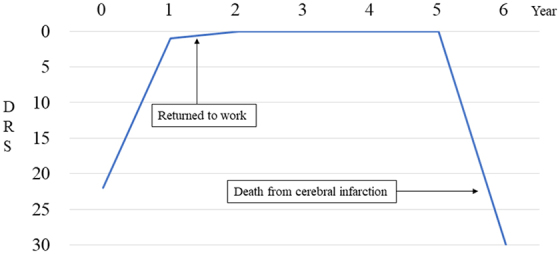
Change in the DRS for case 2. Multiple trauma patient in his twenties, injured in a traffic accident. The patient was at DRS 22 at 1 month post-injury. Six months post-injury, the patient recovered to DRS 1 and was discharged home. One year post-injury, the DRS was 1. At 1 year and 3 months, the patient returned to the same job as before the injury and recovered to DRS 0. However, 6 years post-injury, the patient died because of a stroke. DRS, Disability Rating Scale.

The patient continued to show functional recovery, improving to DRS 1 at 6 years post-injury and has remained at DRS 1 at 14 years post-injury. The item of his DRS scores that remained at 1 was Employability. During this period, he was able to lead a normal social life, including driving a car and getting married. Case 2 was a male in his twenties who suffered from multiple trauma attributable to a traffic accident (Fig. 6). His level of consciousness on hospital arrival was GCS E1V1M5, and head CT showed no obvious intracranial hemorrhage. However, head MRI showed diffuse axonal damage in the dorsal midbrain, corpus callosum, pons, and right basal ganglia. His DRS was 22 at 1 month post-injury. By 6 months post-injury, he had recovered to DRS 1 and was discharged home. His DRS was still 1 at 1 year post-injury. The item of his DRS scores that remained at 1 was Employability. At 1 year and 3 months, the patient returned to the same job as before their injury and recovered to DRS 0. However, 6 years post-injury, the patient died from a stroke.

## Discussion

In this retrospective cohort study, we used the DRS to evaluate long-term outcomes over a 20-year period in patients with so-called persistent VS who remained in a VS at 1 month post-TBI. Patients who remained in a VS after 1 year of injury had a poor outcome thereafter, whereas those who recovered from a VS within 1 year of injury showed improvement in the DRS thereafter.

In 1994, the Multi-Society Task Force on persistent VS defined patients who remain in a VS for more than 1 month post-TBI as being in a persistent VS and those who remain in a VS for more than 1 year as being in a “permanent VS.” Currently, the term permanent VS is not recommended, and it is recommended that the term “chronic VS” be used, accompanied by the duration of VS.^1^ Patients in a VS post-TBI are expected to recover consciousness within 1 year, whereas recovery after 1 year is rare.^2,3^ A systematic review of outcomes of patients in a VS post-TBI also reported that 78% of patients with a persistent VS recovered consciousness by 1 year.^13^ In our study, more than half of patients with a persistent VS recovered from VS within 1 year, which is consistent with previous reports.

In addition, three of the patients in our study also improved from a chronic VS. However, they only improved from a VS to a state of extremely severe disability, which did not contribute to the functional improvement of these patients in the chronic phase. It has been reported that the majority of patients in a chronic VS do not improve from the VS, but a few patients have recovered consciousness,^1^ and some even gained the ability to communicate and participate in social activities.^14^ Another study evaluating the long-term outcomes of TBI patients recovered from VS after 1 year post-injury has shown that significant recovery continues for 2 years post-injury and, to a more modest degree, for 5 years post-injury.^15^ Unfortunately, in this study we were unable to identify any patients in a chronic VS who achieved significant functional recovery as previously reported, with many of these patients having died as an outcome.

Recent studies evaluating the long-term functional outcomes of patients with DoC attributable to TBI, including VS, over a 10-year period have shown that an increasing number of patients achieve functional independence up to 10 years post-injury.^8^ The present study also showed that although improvement in the DRS can be expected for ∼10 years post-injury in patients in a persistent VS who did not progress to a chronic VS, DRS trends plateaued after 10 years and did not appear to improve. We believe that our results strengthen the argument that the functional recovery of patients with DoC attributable to TBI can be expected in the first 10 years, but we did not demonstrate substantial functional recovery beyond the 10th year. We believe that persistent VS patients who recover from VS within 1 year can be expected to gain further improvement in the DRS, which may provide a rationale for continued rehabilitation treatment for patients in a persistent VS.

### Limitations

As one limitation of this study, we were unable to follow the trend of the DRS in 9 patients until the end of the study. In addition, 3 of these 9 patients (including 1 with persistent VS) were lost to follow-up within 10 years, so we could not confirm the long-term follow-up of all 35 patients.

There are also limitations regarding the accuracy of the diagnosis of VS. It is difficult to accurately diagnose VS and evaluate its course by neurobehavioral evaluation, and evaluation using a variety of imaging studies and the Coma Recovery Scale-Revised (CRS-R) is recommended.^16^

Although the CRS-R has also been reported to be useful in differentiating a minimally conscious state (MCS) from a VS,^17^ this study was initiated before such diagnostic criteria were established. Therefore, we used the previously established criteria of diagnosing DRS ≥22 as a VS and defined recovery from a VS if the DRS was ≤21. Therefore, it must be acknowledged that there are limitations regarding the accuracy of the diagnosis of a VS in terms of the accuracy of the differentiation between VS and MCS.

It was not possible to assess exactly what rehabilitation treatment was given to patients in this study. From the DRS survey records, 15 patients were identified as having received rehabilitation treatment, most of whom were non-chronic VS patients. Therefore, we cannot rule out the possibility that there was a difference in rehabilitation treatment intervention between patients who recovered from a VS and those who did not. The effectiveness of rehabilitative treatment for patients in a VS cannot be definitively discussed in this study.

## Conclusion

We followed a cohort of patients who were in a persistent VS at 1 month post-TBI and evaluated their functional outcomes by the DRS up to year 20.

More than half of the patients in a persistent VS at 1 month post-TBI recovered from the VS within 1 year. These patients showed functional improvement for up to 10 years, although a substantial improvement in the DRS beyond that time was not observed. In contrast, most of the chronic VS patients died within 10 years of their injury, and none were alive at 20 years.

## Abbreviations Used

CRS-R: Coma Recovery Scale-Revised
CT: computed tomography
DoC: disorders of consciousness
DRS: Disability Rating Scale
GCS: Glasgow Coma Scale
IQR: interquartile range
MCS: minimally conscious state
MRI: magnetic resonance imaging
TBI: traumatic brain injury
TCDB: Traumatic Coma Data Bank
VS: vegetative state
